# A Reduction Algorithm for Computing The Hybridization Number of Two Trees

**Published:** 2007-05-30

**Authors:** Magnus Bordewich, Simone Linz, Katherine St. John, Charles Semple

**Affiliations:** 1 Department of Computer Science, Durham University, Durham DH1 3LE, United Kingdom; 2 Biomathematics Research Centre, Department of Mathematics and Statistics, University of Canterbury, Christchurch, New Zealand; 3 Department of Bioinformatics, Heinrich-Heine-University, Düsseldorf, Germany; 4 Department of Mathematics and Computer Science, Lehman College, City University of New York, USA

**Keywords:** Hybridization networks, reticulate evolution, agreement forest

## Abstract

Hybridization is an important evolutionary process for many groups of species. Thus, conflicting signals in a data set may not be the result of sampling or modeling errors, but due to the fact that hybridization has played a significant role in the evolutionary history of the species under consideration. Assuming that the initial set of gene trees is correct, a basic problem for biologists is to compute this minimum number of hybridization events to explain this set. In this paper, we describe a new reduction-based algorithm for computing the minimum number, when the initial data set consists of two trees. Although the two-tree problem is NP-hard, our algorithm always gives the exact solution and runs efficiently on many real biological problems. Previous algorithms for the two-tree problem either solve a restricted version of the problem or give an answer with no guarantee of the closeness to the exact solution. We illustrate our algorithm on a grass data set. This new algorithm is freely available for application at either http://www.bi.uni-duesseldorf.de/~linz or http://www.math.canterbury.ac.nz/~cas83.

## 1. Introduction

Evolutionary (phylogenetic) trees are used to represent the tree-like evolution of a collection of present-day species. For many groups of taxa (for example, most mammals), this is an appropriate representation. However, because of non-tree-like evolutionary processes such as hybridization, horizontal gene transfer, and recombination, not all groups of taxa are suited to this type of representation. Collectively referred to as reticulation events, these processes result in species that are a mixture of DNA regions derived from different ancestors.

In the following, we restrict our attention to hybridization. During such an event, two lineages recombine to create a new species which may have the same number of chromosomes as its parents (diploid hybridization) or the sum of all parental chromosomes (polyploid hybridization). Eukaryotes, whose evolutionary past contains hybridization, include certain groups of plants, birds, and fish (see Mallet, 2005).

The effect of hybridization in evolution has been recognized for quite some time. For example, since 1930’s, botanists have suggested that the morphological variation in the New Zealand flora is due to hybridization (Allan, 1961). However, the computational task of determining how much hybridization has occurred has been a much more recent consideration. In regards to this task, a fundamental problem for the biologists studying the evolution of species whose past includes hybridization is the following: given a collection of rooted phylogenetic trees on sets of species that correctly represents the tree-like evolution of different parts of their genomes, what is the smallest number of hybridization events needed to explain the evolution of the species under consideration. As well as providing a lower bound on the number of such events, this smallest number also provides an indicator for the extent to which hybridization has influenced the evolutionary history of the considered collection of present-day species.

Formalized mathematically, this fundamental problem is NP-hard even when the initial collection consists of two rooted binary phylogenetic trees (Bordewich and Semple, 2007a). Consequently, as a result of this computational difficulty, most current research considers the two-tree problem. Now there are several algorithms for approaching this latter problem. However, all these algorithms are either algorithms solving a restricted version of the problem (e.g. Hallett and Lagergren, 2001; Huson et al. 2005; Nakhleh et al. 2005b) or polynomial-time heuristics with no guarantee of the closeness of their solution (e.g. Nakhleh et al. 2005a).

In this paper, we describe a new, and recently implemented, exact algorithm for solving the two-tree problem (with no restrictions) based on three reductions that preserve the amount of hybridization. All of these reductions make use of the similarities between the two trees. It has been recently shown that two of the reductions are enough to guarantee that the algorithm is fixed-parameter tractable, where the parameter is the smallest number of hybridizations to explain the initial two trees (Bordewich and Semple, 2007b). This means that the algorithm runs efficiently when this smallest number is bounded. The remaining reduction allows for a divide-and-conquer approach when the two trees share common clusters.

The new algorithm described in this paper has been implemented in Perl and is available for application at http://www.bi.uni-duesseldorf.de/~linzand http://www.math.canterbury.ac.nz/~cas83. As the implementation requires the two input trees to be given in a special type of string format, the interested reader can also download two sample trees and a short manual describing how to use the software. The program output contains the simplified trees after applying the three reductions (see Section 2.1) and the minimum number of hybridization events to explain the two initial trees.

The notation and terminology in this paper follows Semple and Steel (2003). The paper is organized as follows. In the next section, we formalize the problem, describe the three reductions, and outline the algorithm. As the two-tree problem is NP-hard, there are going to be some instances for which the algorithm will not return an answer in a reasonable time—in particular, instances that have a high level of hybridization and few similarities. Nevertheless, there are many instances for which the algorithm performs exceptionally well. In terms of their running time, a full range of instances are highlighted in Section 3, where we apply the algorithm to a grass (*Poaceae*) data set which consists of sequence data for six genetic loci and six corresponding gene trees. Each of the 15 different pairs of trees are considered.

Full details of the algorithm described in this paper can be found in Appendix A, where a pseudocode version is given. The algorithm is a combination of the fixed-parameter result as described in Bordewich and Semple, (2007b) (whose proof of correctness is given by Proposition 3.2 of that paper) and the cluster reduction described in Baroni et al. (2006) (whose proof of correctness is given by Theorem 1 in that paper). For simplicity, in this paper, we only describe the main ideas. The reader who is interested in the finer details, we refer them to the original papers.

## 2. Reduction Algorithm for Hybridization

We begin with a formal description of the two-tree problem. A *rooted binary phylogenetic X*-*tree* T is a rooted tree that has leaf set *X* and whose root has degree two while all other interior vertices have degree three. A *cluster* of T is a subset of *X* that contains precisely the elements that are descendants of some vertex of T.

A *rooted acyclic digraph* is a digraph with no directed cycles. Each such digraph has a distinguished vertex *ρ* whose in-degree is zero and has the property that there is a directed path from *ρ* to every other vertex. For a vertex υ in a digraph, we will denote the *in-degree* of υ (the number of edges directed into υ) by *d* ^−^(υ) and the *out-degree* of υ (the number of edges directed out of υ) by *d* ^+^(υ). A *hybridization network* H on *X* is a rooted acyclic digraph with root *ρ,* in which

- *X* is the set of vertices of out-degree zero,
- *d* ^+^(*ρ*) ≥2, and
- for all other vertices υ, *d* ^−^(υ) ∈{1,2}, and no vertex υ has *d* ^−^(υ) = 1 and *d* ^+^(υ) = 1.

**Figure 1.**
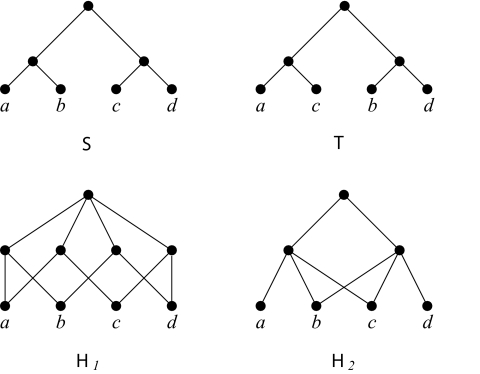
Two rooted binary phylogenetic trees S and T and the two hybridization networks ${\text{H}}_{1}$ and ${\text{H}}_{2}$ which explain both trees.

To illustrate these concepts, two rooted binary phylogenetic trees S and T and two hybridization networks ${\text{H}}_{1}$ and ${\text{H}}_{2}$ are shown in Figure 1. In all cases,

X = {a,b,c,d}.

Analogous to rooted binary phylogenetic *X*-trees, hybridization networks on *X* can be used to represent the ancestral history of a collection of present-day species that includes hybridization. The set *X* represents the collection of present-day species. Vertices of in-degree two represent an exchange of genetic information between the hypothetical ancestors. These vertices are called as *hybridization vertices*. To quantify the number of hybridization events, the *hybridization number* of a hybridization network H, denoted as *h*( H), is the number of hybridization vertices. In Figure 1, *h*( ${\text{H}}_{1}$) = 4 and *h*( ${\text{H}}_{2}$) = 2, respectively. Note that the hybridization vertices need not always appear at the ‘tips’ of a network. Furthermore, observe that rooted binary phylogenetic trees are special types of hybridization networks. As one would expect, the hybridization number of such a network is zero.

Let T be a rooted binary phylogenetic *X*-tree and H be a hybridization network on *X*. We say that H *explains* T if all of the ancestral relationships described in T are covered by H. Mathematically speaking, H explains T if T can be obtained from H by deleting a subset of the edges of H together with any resulting isolated vertices and suppressing any degree-two vertex. For example, both ${\text{H}}_{1}$ and ${\text{H}}_{2}$ explain each of S and T in Figure 1. For two rooted binary phylogenetic *X*-trees S and T, let *h*( S, T) denote the smallest number of hybridization vertices over all hybridization networks that simultaneously explains S and T. Referring to Figure 1, it is easily checked that at least two hybridization events are needed to explain S and T. Since *h*( ${\text{H}}_{2}$) = 2, it follows that *h*( S, T) = 2. Given two rooted binary phylogenetic *X*-trees S and T, the two-tree problem is to find *h*( S, T). For convenience, we refer to this problem as the Hybridization Number problem.

Called HybridNumber, the new algorithm described in this paper finds the solution to Hybridization Number. We briefly describe next about a combinatorial characterization of *h*( S, T). This characterization underlies HybridNumber. Loosely speaking, a *forest* of S (or T) is a collection of non-overlapping rooted subtrees of S (or T) whose (disjoint) union of leaf sets is *X*. An *agreement* forest F of S and T is a forest of both S and T. Beginning with a hybridization network that explains S and T, one way to obtain an agreement forest for S and T is by deleting each of the edges coming into every hybridization vertex. Biologically, the deleted edges correspond to different paths of genetic inheritance. Thus, the fewer the number of hybridization vertices of such a network, the smaller the size of the resulting agreement forest for S and T, where the size of a forest is the number of trees in the forest. On the other hand, if we are given an agreement forest for S and T, then one can reverse this process to construct a hybridization network H that explains S and T provided the forest has a particular acyclicity property. This property excludes the possibility of circular inheritance which means that a vertex in H does not inherit genetic information from its own descendants, in which case H contains no directed cycles. An agreement forest with the acyclicity property is called *acyclic*. Theorem 2 of Baroni et al. (2005) showed that *h*( S, T) is one less than the minimum size of an acyclic-agreement forest for S and T.

The algorithm HybridNumber is based on the repeated use of three polynomial-time reduction rules. Essentially, each of these rules preserves the hybridization number in some way. The first two rules, ‘subtree’ and ‘chain’ reduction, reduce the size of the problem instance, while the third rule, ‘cluster’ reduction breaks the problem into a number of smaller and more tractable problems. An exhaustive search part on each of the smaller problems completes the algorithm. While it is likely that the general problem HybridNumber has no polynomial-time solution, it would be interesting to see how one could speed up the last part of the algorithm.

### 2.1. Reductions

**Figure 2.**
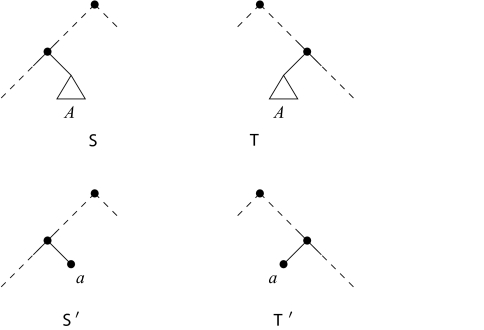
Two rooted binary phylogenetic trees S and T reduced under the subtree reduction rule. The triangle *A* indicates a maximal subtree which is common to both trees and this is replaced by the new leaf labeled *a* in ${\text{S}}^{\prime }$ and ${\text{T}}^{\prime }$

**Figure 3.**
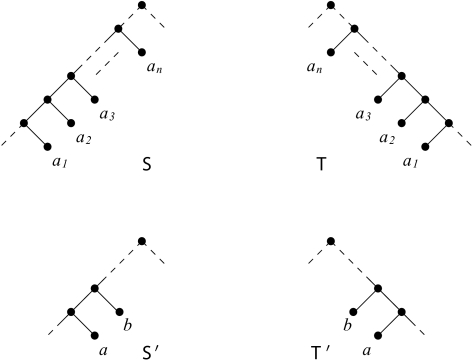
Two rooted binary phylogenetic trees S and T reduced under the chain reduction rule.

**Figure 4.**
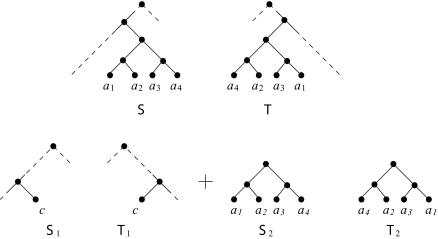
Two rooted binary phylogenetic trees S and T divided under the cluster reduction rule. The hybridization number of S and T is the sum of the hybridization numbers of $S_{1}$ and $T_{1}$, and $S_{2}$ and $T_{2}$.

In this subsection, we describe the three reductions and their effects on computing *h*( S, T) for two rooted binary phylogenetic *X*-trees S and T. The reductions are illustrated in Figures 2, 3, and 4, respectively. Pseudocode for each of the three reduction rules can be found in Appendix A.

1. *Subtree reduction*: Replace a maximal pendant subtree with at least two leaves that occurs identically in S and T by a single leaf with a new label. If ${\text{S}}^{\prime }$ and ${\text{T}}^{\prime }$ denote the resulting trees, then
  $$h(\text{S},\;\text{T})\;=\;h({\text{S}}^{\prime },\;{\text{T}}^{\prime }).$$
2. *Chain reduction*: Replace a maximal chain of at least three leaves that occur identically and with the same orientation relative to the root in S and T by two new leaves with new labels, *a* and *b* say, correctly orientated to preserve the direction of the chain. If the chain consists of *n* leaves, then assign the pair {*a*,*b*} of new leaves weight *n* – 2. If ${\text{S}}^{\prime }$ and ${\text{T}}^{\prime }$ denote the resulting trees, then either
  $$h(\text{S},\;\text{T})\;=\;h({\text{S}}^{\prime },\;{\text{T}}^{\prime }),$$
  or
  $$h(\text{S},\;\text{T})\;=\;h({\text{S}}^{\prime },\;{\text{T}}^{\prime })\;+\;(n\;-\;2),$$
  depending on whether a minimum-size acyclic-agreement forest for ${\text{S}}^{\prime }$ and ${\text{T}}^{\prime }$ has the property that *a* and *b* are in the same subtree or not, respectively. In the case that *a* and *b* are not in the same subtree, *a* and *b* are isolated vertices in the minimum-size acyclic-agreement forest (Bordewich and Semple, 2007b). The effect of this is that, in a minimum-size acyclic-agreement forest for S and T, each of *a_1_*, *a_2_*,..., *a_n_* are isolated. The purpose of the weighting is to keep track of the number of such vertices when *a* and *b* are isolated.
  There is a slight complication here in that the reducing chain may contain consecutive pairs of leaves that have previously been involved in a chain reduction. In such cases, the pair {*a*, *b*} of new leaves is assigned a weight that is the sum of the associated weights of these pairs and *n* – 2. The effect on *h*( S, T) is a generalization of the previous outcome.
3. *Cluster reduction*: If *A* is a minimal cluster common to S and T and with at least two leaves, then replace S and T with two pairs of new trees. The first pair, ${\text{S}}_{1}$ and ${\text{T}}_{1}$ say, are the subtrees of S and T whose leaf set is *A*, while the second pair, ${\text{S}}_{2}$ and ${\text{T}}_{2}$ say, are obtained from S and T by replacing the subtrees whose leaf set is *A* with a new label. The point of this is that
  $$h(\text{S},\;\text{T})\;=\;h({\text{S}}_{1},{\text{T}}_{1})\;+\;h({\text{S}}_{2},\;{\text{T}}_{2}).$$

### Remarks

- The fact that the cluster reduction rule, and consequently the subtree reduction rule, preserve the number of hybridization events in the way that is described above, is shown in Theorem 1 of Baroni et al. (2006). Furthermore, the correctness of the chain reduction rule follows from Proposition 3.2 of Bordewich and Semple (2007b).
- Bordewich and Semple (2007b) showed that the subtree and chain reductions by themselves are enough to ‘kernelize’ the problem and give a fixed-parameter algorithm for Hybridization Number. The cluster reduction provides an extremely useful tool for breaking the problem into a number of smaller problems—all that is required is that the subtrees should have identical leaf sets, the topologies of the two subtrees can be completely different.
- Without going into details, the cluster reduction has a similar flavor to the “Decomposition Theorem” in Huson et al. (2005). This theorem describes a one-to-one correspondence between the overlapping cycles of an (unrooted) network N, the connected components of the incompatibility graph of the splits generated by N, and the netted components of the splits graph of the splits generated by N. However, while this theorem yields an algorithm for minimizing the number of hybridization vertices amongst a restricted class of networks, it is important to note that it does not give a general strategy for minimizing this number amongst all hybridization networks as there is no guarantee that such a reduction leads to an optimal solution. In contrast, Baroni et al. (2006) showed that such a strategy, in particular the cluster reduction, works for two trees. It is an interesting open problem whether this extends to more than two trees. An analogous problem has also been posed by Gusfield and Bansal (2005) within the framework of population genetics.

Using the three reduction rules, the algorithm HybridNumber initially attempts to reduce the size of the problem instance as much as possible. It begins by repeatedly applying the subtree reduction where possible before applying the chain reduction in the same way. Once this is done, it finds the smallest common cluster of size at least two of the resulting trees and uses this cluster to perform a cluster reduction, thus replacing the pair of subtree-and-chain-reduced trees with two smaller pairs of trees. Putting aside the pair of trees corresponding to the common cluster, the algorithm now repeats this process for the other pair of trees. Eventually, no more reductions are possible and we are left with pairs of trees for which we exhaustively find each of their hybridization numbers. Because of the combinatorial characterization mentioned earlier, up to the weightings resulting from a chain reduction, this exhaustive process finds an acyclic-agreement forest of smallest size for each pair of trees. The sum of these sizes gives the hybridization number of the initial two trees.

## 3. The Grass (*Poaceae*) Data Set

In this section, we describe the application of HybridNumber to a grass (*Poaceae*) data set. This data set was provided by the Grass Phylogeny-Working Group (2001). Although the extent of hybridization is still discussed controversially (Rieseberg et al. 2003), the occurrence of such events in certain groups of plants is generally accepted. In 1996, Ellstrand et al. examined the frequency of spontaneous hybridization in five biosystematic floras and found that, in four of these floras, the *Poaceae* family is among the six families with the highest number of natural hybrids. Therefore, it is more likely that the conflicting signals in the data are due to hybridization rather than other factors and so it is an appropriate data set for our purposes.

The *Poaceae* data set consists of sequence data for six different genetic loci: internal transcribed spacer of ribosomal DNA (*ITS*); NADH dehydrogenase, subunit F (*ndhF*); phytochrome B (*phyB*); ribulose 1,5-biphosphate carboxylase/oxygenase, large subunit (*rbcL*); RNA polymerase II, subunit β” (*rpoC2*); and granule bound starch synthase I (*waxy*). A summary describing the sequence origin, the number of sequences, and the alignment length for each gene in the data set is given in Table 1.

For each loci, a rooted binary phylogenetic tree was reconstructed using the fastDNAmL program (Olsen et al. 1994). These gene trees were supplied by Heiko Schmidt who has previously analyzed this data set (Schmidt, 2003). We (separately) applied HybridNumber to each of the 15 different pairwise combinations of gene trees, where, for each combination, we restricted the gene trees to taxa common to both. The size of the overlapping taxa set for each combination is given in the second column of Table 2.

**Figure 5.**
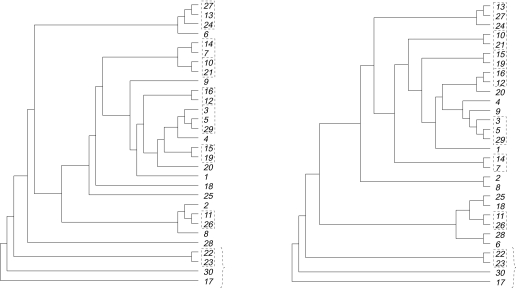
The input to HybridNumber for the combination *phyB* and *ITS*. Restricting to overlapping taxa, the tree resulting from the nuclear sequence *ITS* is on the left, while the tree resulting from the chloroplast sequence *phyB* is on the right. Labels in boxes denote the eight maximal pendant subtrees that are common to both trees, and the brace denotes a maximal chain once we have applied the subtree reductions.

Before detailing the contents of Table 2, we describe one particular application of Hybrid-Number that highlights the extent to which the reductions incorporated in HybridNumber can reduce the size of the problem instance. This application involves the two phylogenetic trees of the chloroplast sequence phytochrome B (*phyB*) and the nuclear sequence of the internal transcribed spacer of ribosomal DNA (*ITS*) which have an overlapping taxa set of 30 present-day species (see the row indicated by the gray background in Table 2). These two trees with the restricted taxa set are shown in Figure 5. To enable a reader-friendly presentation of both trees, we have replaced the correct species names by numbers.

**Figure 6.**
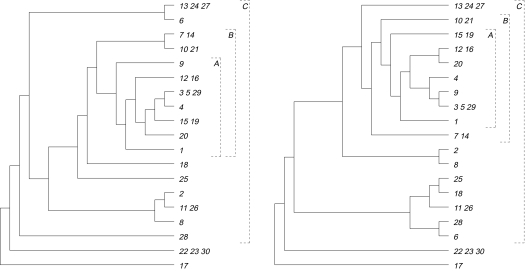
The two resulting phylogenetic trees (left: *ITS*, right: *phyB*) after repeated applications of the subtree reduction and then the chain reduction to the two trees in Figure 5. The three brackets *A*, *B*, and *C* indicate common clusters.

Taking the two trees, in Figure 5, as input to HybridNumber, the algorithm initially finds all maximal pendant subtrees that are common to both trees (indicated by small boxes in Figure 5) and replaces each such subtree with a single leaf whose label is a concatenation of the subtree labels. Here there are eight such subtrees. Next, Hybrid-Number checks for any identical chains of leaves in the two resulting trees. There is one such maximal chain of leaves and this is denoted by the brace in Figure 5. Applying the chain reduction, the labeling of the species which has evolved first is kept, while the labels of all other chain leaves are concatenated. The two trees resulting from the subtree and chain reductions are shown in Figure 6.

**Figure 7.**
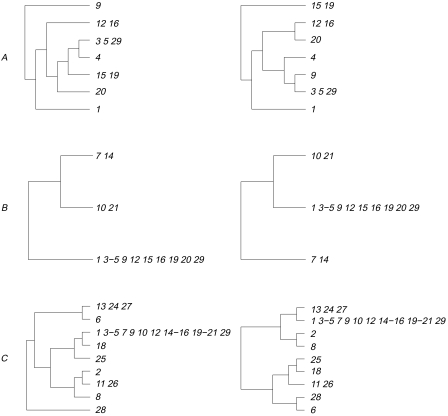
The three pairs of clusters *A*, *B*, and *C* corresponding to Figure 6 for which HybridNumber (separately) calculates the minimum number of hybridization events (left: *ITS*, right: *phyB*).

In the next step, the cluster reduction rule divides the problem into two smaller problems by searching for a minimal cluster of size at least two that is common to both trees in Figure 6. The first such cluster, shown by square bracket *A* in Figure 6, is {{9}, {12, 16}, {3, 5, 29}, {4}, {15, 19}, {20}, {1}} and the corresponding subtrees are shown at the top of Figure 7. At this point, HybridNumber has completed one iteration. Beginning with the two trees that result from replacing the cluster shown by *A* with a single new leaf (a concatenation of the leaves in the cluster), the algorithm performs two further iterations. At the end of these two iterations, we obtain two more pairs of trees as indicated by the square brackets *B* and *C* in Figure 6. These two pairs are shown in Figure 7. At this stage, the original inputted trees have been reduced to two identical trees.

The final step in the algorithm is to exhaustively find the hybridization number of the three pairs of non-identical trees in Figure 7. The first pair has hybridization number 3, while the second and third pairs have hybridization numbers of 1 and 4, respectively. Adding the three numbers together gives the hybridization number of 8 for the two trees shown in Figure 5. The running time of this particular application is about 19 seconds (see Table 2).

This is remarkably quick given that the two initial trees contain 30 taxa and the hybridization number is 8. As a comparison, we tried finding the hybridization number of these two trees without the three reductions. After 1 week, the algorithm was still running!

In Table 2, the results for all 15 pairs of trees are summarized. The running times are given in days, hours, or seconds. For eight pairs, HybridNumber calculates the hybridization number within a couple of minutes. Furthermore, the hybridization numbers of all but three pairs are found within a time span of 2 days. The successfully completed pairs contained up to 40 taxa and have hybridization numbers as high as 14. Those three pairs of trees for which the running time is given as 2 days in Table 2 are instances of the described NP-hard problem for which the algorithm will not return an answer in reasonable time. Nevertheless, we still have a lower bound on their respective hybridization numbers depending upon the intermediate result of the algorithm after 2 days at which time we stopped the algorithm. Lastly, the difference in running times of the various pairs is due to the extent of the reductions that we were able to use to reduce the problem instance and their hybridization number if the reductions have little effect. (The running time is dependent on the exhaustive search part of the algorithm as the reductions take a matter of seconds.) However, it is worth noting that it is always possible to reduce the number of leaves in a pair of trees to a linear function of its hybridization number (Bordewich and Semple, 2007b)—again highlighting the effectiveness of the reductions.

## 4. Conclusion

Due to reticulate evolution, phylogenetic gene trees reconstructed for different genetic loci often reveal conflicting tree topologies, because processes like hybridization, horizontal gene transfer, and recombination are not tree-like. The extent to which such events occur is of increasing interest for many evolutionary studies.

In this paper, we have described a newly implemented algorithm to calculate exactly the minimum number of hybridization events that explains two phylogenetic gene trees. Unlike previous algorithms, HybridNumber is not a heuristic, and its solution is not restricted in any way. Calculating this minimum number is computationally a hard problem, and so if the initial two gene trees only share a few similarities, then in many cases the exact calculation of the hybridization number is computationally infeasible. However, if the two gene trees share a number of common features—pendant subtrees, chains, or clusters—which is likely for many biological examples, the new algorithm performs remarkably well and the hybridization number can be found in reasonable time.

Note that HybridNumber calculates a lower bound for the number of hybridization events to explain the differences between two phylogenetic gene trees (assuming that hybridization is the only cause of incongruence between the two trees). It is possible that the real number of hybridization events that happened during the evolution of the collection of present-day species under consideration is underestimated. Indeed, it is possible that some hybridization events are never recognized. Nevertheless, the algorithm provides an important first step towards an understanding of the extent to which hybridization has influenced evolution.

Of course, in addition to computing the hybridization number of two rooted phylogenetic *X*-trees S and T, one is also interested in constructing hybridization networks that realize this number. This can be efficiently done from a minimum-sized acyclic-agreement forest F for S and T. Intuitively, one takes the tree in F containing the root of S and T, and then systematically adjoins the rest of the trees in F as follows. At each step, adjoin a tree from F whose root is not the descendant (relative to either S or T) of any tree not already adjoined. Each tree in F is adjoined with two edges to the current hybridization network so that the resulting hybridization network explains the appropriate restrictions of S and T.

Finally, it is clear that extending this work to allow for more than two trees in the input is important. Such extensions are discussed in the corresponding author’s PhD thesis.

## Figures and Tables

**Table 1.** The *Poaceae* data set.

**Loci**	**Sequence origin**	**# Sequences**	**Alignment length**
*ITS*	Nucleus	47	322
*ndhF*	Chloroplast	65	2210
*phyB*	Nucleus	40	1182
*rbcL*	Chloroplast	37	1344
*rpoC2*	Chloroplast	34	777
*waxy*	Nucleus	19	773

**Table 2.** Results for the *Poaceae* data set.

**Pairwise combination**		**# Taxa**	**Hybridization number**	**Run time^a^**
*ndhF*	*phyB*	40	14	11 h
*ndhF*	*rbcL*	36	13	11.8 h
*ndhF*	*rpoC2*	34	12	26.3 h
*ndhF*	*waxy*	19	9	320 s
*ndhF*	*ITS*	46	At least 15	2 d
*phyB*	*rbcL*	21	4	1 s
*phyB*	*rpoC2*	21	7	180 s
*phyB*	*waxy*	14	3	1 s
*phyB*	*ITS*	30	8	19 s
*rbcL*	*rpoC2*	26	13	29.5 h
*rbcL*	*waxy*	12	7	230 s
*rbcL*	*ITS*	29	At least 9	2 d
*rpoC2*	*waxy*	10	1	1 s
*rpoC2*	*ITS*	31	At least 10	2 d
*waxy*	*ITS*	15	8	620 s

a Run time on a 2000 MHz CPU, 2 GB RAM machine measured in seconds (s), hours (h), and days (d), respectively.

## Appendix A

### Pseudocode

Here, we present the pseudocode of HybridNumber. For a rooted binary phylogenetic *X-*tree T and a subset *A* of *X*, we denote the minimal subtree of T connecting the elements in *A* by T (*A*). Further, we denote the tree formed by replacing a cluster *A* with the new leaf *c* by T [*A* → *c*]. If *B* is a subset of *X*, we use T [ *B*] to denote the phylogenetic tree obtained from T by deleting each of the elements in *B* and suppressing any resulting degree-two vertex. Finally, F( T, *E*) denotes the forest obtained from the tree T by deleting the edges in the set *E*. Because of the chain reduction rule, the input to HybridNumber includes a weight function *w* on pairs of taxa; this can be taken to be zero for all pairs in the initial input.

**Algorithm A.1**: HybridNumber ( S, T, *w*)

( S, T, *w*) ← SubtreeReduction ( S, T, *w*)

( S, T, *w*) ← ChainReduction ( S, T, *w*)

**if** ∃ a minimal common cluster *C* of S and T **and** 1 < |C| < number of taxa of S

$$\text{do}\;\{\begin{array}{l} \left({\text{S}}_{1},\;{\text{T}}_{1},\;w_{1},\;{\text{S}}_{2},\;{\text{T}}_{2},\;w_{2})\;\leftarrow \;\text{ClusterReduction}\;(\text{S},\;\text{T},\;w\right)\\ h_{1}\;\leftarrow \;\text{ExhaustiveSearch}\;({\text{S}}_{1},\;{\text{T}}_{1},\;w_{1})\\ h_{2}\;\leftarrow \;\text{HybridNumber}({\text{S}}_{2},\;{\text{T}}_{2},\;w_{2})\\ h\;\leftarrow \;h_{1}\;+\;h_{2} \end{array}$$

  **else**

  **do** *h* ← ExhaustiveSearch ( S, T, *w*)

**return** (*h*)

**Algorithm A.2:** SubtreeReduction ( S, T, *w*)

*A* ← maximal common subtree of S and T

**if** |*A*| > 1

$$\text{do}\;\{\begin{array}{l} {\text{S}}^{\prime }\;\leftarrow \;\text{S}[A\;\to \;a]\\ {\text{T}}^{\prime }\;\leftarrow \;\text{T}[A\;\to \;a]\\ \left(\text{S},\;\text{T},\;w)\;\leftarrow \;\text{SubtreeReduction}\;\;({\text{S}}^{\prime },\;{\text{T}}^{\prime },\;w\right) \end{array}$$

**return** ( S, T, *w*)

**Algorithm A.3:** ChainReduction ( S, T, *w*)

(*a**_1_*, …, *a**_n_*) ← maximal common chain of S and T

**if** *n* ≥ 3

$$\text{do}\;\{\begin{array}{l} weight\;\leftarrow \;\sum_{i=1}^{n-1}w\;(a_{i},\;a_{i+1})\\ w(a,\;b)\;\leftarrow \;weight\;+\;(n\;-\;2)\\ {\text{S}}^{\prime }\;\leftarrow \;\text{S}[\{a_{1}\}\;\to \;a,\;\{a_{2}\}\;\to \;b,\;-\;\{a_{3},\;\ldots \;,\;a_{n}\}]\\ {\text{T}}^{\prime }\;\leftarrow \;\text{T}[\{a_{1}\}\;\to \;a,\;\{a_{2}\}\;\to \;b,\;-\;\{a_{3},\;\ldots \;,\;a_{n}\}]\\ w^{\prime }\;\leftarrow \;\{w(a,\;b)\}\;\cup \;w\;\text{restricted}\;\text{to}\;\text{pairs}\;\text{not}\;\text{in}\{a_{1},\;\ldots \;,\;a_{n}\}\\ \left(\text{S},\;\text{T},\;w)\;\leftarrow \;\text{ChainReduction}({\text{S}}^{\prime },\;{\text{T}}^{\prime },\;w^{\prime }\right) \end{array}$$

**return** ( S, T, *w*)

**Algorithm A.4:** ClusterReduction ( S, T, *w*)

*C* ← minimal common cluster of S and T

${\text{S}}_{1}$ ← S(*C*)

${\text{S}}_{2}$ ← S[*C* →*c*]

${\text{T}}_{1}$ ← T(*C*)

${\text{T}}_{2}$ ← T [*C* → *c*]

*w*_1_ ← *w* restricted to pairs of taxa in *C*

*w*_2_ ← *w* restricted to pairs of taxa not in *C*

**return** ( ${\text{S}}_{1}$, ${\text{T}}_{1}$, *w*_1_, ${\text{S}}_{2}$, ${\text{T}}_{2}$, *w*_2_)

**Algorithm A.5:** ExhaustiveSearch ( S, T, *w*)

**if** S≅ T **return** (0)

*h* ← number of leaves of S

*i* ← 0

**repeat**

  **for each** *E* a subset of the edges of S such that |*E*| = *i*

$$\text{do}\;\{\begin{array}{l} \text{F}\;\leftarrow \;\text{F}(\text{S},\;E)\\ if\;\text{F}\;\text{is}\;\text{an}\;\text{acyclic}-\text{agreement}\;\text{forest}\;\text{of}\;\text{S}\;\text{and}\;\text{T}\\ \text{do}\;\{\begin{array}{l} P\;\leftarrow \;\left\{(a,\;b):\;a,b\;\text{are}\;\text{isolated}\;\text{taxa}\;\text{in}\;\text{F}\right\}\\ h^{\prime }\;\leftarrow \;i\;+\;\sum_{(a,b)\in P}w(a,\;b)\\ \text{if}\;h^{\prime }\;<\;h\\ \;\;\;\text{do}\;h\;\leftarrow \;h^{\prime } \end{array} \end{array}$$

*i* ← *i+*1

**until** *i* ≥*h*

**return** (*h*)

### Remarks

1. The actual implemented algorithms contain various small improvements compared to the pseudocode in order to improve the running time. Whilst these changes do not affect the theoretical ‘worst case’ running time, in practice they are beneficial. An example is that no agreement forest has an isolated internal vertex, hence in the exhaustive search we do not need to consider subsets of edges of size *i* (to delete from S) which contain the three edges incident with a particular vertex.
2. In HybridNumber, following a call to the cluster reduction, the cluster removed cannot be reduced further using the reductions, in which case we immediately call ExhaustiveSearch. However, it may now be possible to further reduce the remainder of the trees and so we call HybridNumber.
3. In ExhaustiveSearch, if we have found a forest of weight *h* formed by deleting fewer than *h* edges, we must run until we have checked all possible forests resulting from the deletion of up to *h* edges in case there exists one of lower weight. This check is a consequence of the way in which the chain reduction works.
